# Self-Reported Serious Illnesses in Rural Cambodia: A Cross-Sectional Survey

**DOI:** 10.1371/journal.pone.0010930

**Published:** 2010-06-03

**Authors:** Por Ir, Chean Men, Henry Lucas, Bruno Meessen, Kristof Decoster, Gerald Bloom, Wim Van Damme

**Affiliations:** 1 Provincial Health Department, Ministry of Health, Siem Reap, Cambodia; 2 Department of Public Health, Institute of Tropical Medicine, Antwerp, Belgium; 3 Centre for Advanced Studies, Phnom Penh, Cambodia; 4 Institute of Development Studies, University of Sussex, Brighton, United Kingdom; Erasmus University Rotterdam, Netherlands

## Abstract

**Background:**

There is substantial evidence that ill-health is a major cause of impoverishment in developing countries. Major illnesses can have a serious economic impact on poor households through treatment costs and income loss. However, available methods for measuring the impact of ill-health on household welfare display several shortcomings and new methods are thus needed. To understand the potential complex impact of major illnesses on household livelihoods, a study on poverty and illness was conducted in rural Cambodia, as part of an international comparative research project. A cross-sectional survey was performed to identify households affected by major illness for further in-depth interviews.

**Methodology and Principal Findings:**

5,975 households in three rural health districts were randomly selected through a two-stage cluster sampling and interviewed. 27% of the households reported at least one member with a serious illness in the year preceding the survey and 15% of the household members reported suffering from at least one serious illness. The most reported conditions include common tropical infectious diseases, chronic diseases (notably hypertension and heart diseases) and road traffic accidents. Such conditions were particularly concentrated among the poor, children under five, women, and the elderly. Poor women often reported complications related to pregnancy and delivery as serious illnesses.

**Conclusions and Significance:**

Despite some methodological limitations, this study provides new information on the frequency of self-reported serious illnesses among the rural Cambodia's population, which serves as a basis for further in-depth investigation on ‘major illnesses’ and their economic consequences on poor households. This can in turn help policy makers to formulate appropriate interventions to protect the poor from the financial burden associated with ill-health. Our findings suggest that every year a considerable proportion of rural population in Cambodia, especially the poor and vulnerable, are affected by serious illnesses, both communicable and non-communicable diseases.

## Introduction

Poverty correlates with ill-health, creating a vicious circle: poverty breeds ill-health and ill-health often exacerbates poverty, especially in the absence of effective social health protection. Poor people tend to have worse health and suffer more frequently from severe health problems than the better-off do [1], [2]. Serious illness does not only cause suffering and death, but also has an important financial cost; direct out-of-pocket payments for treatment and illness-related income loss can make a non-poor household poor and push a poor household into deeper poverty [3], [4]. There is substantial evidence that ill-health is a major cause of impoverishment, especially in countries where public funding for health services is insufficient and social health protection schemes are underdeveloped or unavailable [5]–[7].

Despite considerable improvements in the health sector, access to affordable and effective health care remains a problem in Cambodia, especially for the poor and vulnerable population. Two thirds of total health expenditure is paid directly out-of-pocket by users, mainly in the private sector. When people require health care, they can choose from a wide variety of health care providers, including government health centres and hospitals, private for-profit and not-for-profit clinics and hospitals, private pharmacies, informal drug vendors, and traditional healers [8]. Most often, patients or caretakers ‘shop around’ in search of treatment and pay considerable amounts of money directly out-of-pocket, which can lead to asset depletion and indebtedness, thereby jeopardizing the future welfare of their households [9]–[11].

Considerable progress in measuring the impact of ill-health on household welfare has been made over the last years, but there are still knowledge gaps. To develop appropriate policies to protect households against impoverishing effects of ill-health, an understanding of the multiple and complex pathways by which ill-health affects wellbeing is required. Several key concepts have been put forward in the literature, including ‘health shocks’, ‘major illnesses’, and ‘catastrophic health expenditures’. The latter, which refers to situations in which health expenditures exceed a given percentage of disposable income, has several limitations. This measurement is inaccurate when households finance a substantial share of health expenditures through coping strategies, such as borrowing [12], [13]. Another shortcoming of this method may be the fact that poor people often spend very little on health care due to their inability or unwillingness to pay for it, given other demands on their extremely limited resources. Measuring incidence of catastrophic health expenditures often relies on large-scale surveys, which do not provide much qualitative insight and lack a social dimension, and this gap can be filled in by in-depth case studies [14]. Therefore, other methods, which combine a rapid cross-sectional survey to identify households suffering potentially ‘catastrophic’ events and in-depth studies on the affected households, are needed to measure the impact of ill-health on household welfare and poverty [15].

Many common illnesses and injuries are relatively mild and can be easily cured with little cost. These are considered ‘minor illnesses’. However, effects of some diseases can be more profound: they cause anxiety, last long, fail to respond to treatment, have high treatment costs and undermine income-generating ability, and thus increase the risk of impoverishment. In such cases, the household may report the condition as a ‘major illness’. To understand the potential complex impact of ‘major illness’ on household livelihoods, EC-funded international research on Poverty and Illness (POVILL: www.povill.com) conducted a comparative study in three countries: Cambodia, China and Laos. A reasonably large number of households apparently affected by ‘major illness’ were selected through a cross-sectional household survey that used a strict probability sampling method, the so-called Rapid Household Survey (RHS). We report the findings from the RHS in Cambodia with an emphasis on the frequency of self-reported ‘serious illnesses’ and their distribution across socio-economic, education, gender, and age groups, as a preparatory step for further analysis.

## Methods

### Study setting

The Cambodian health system follows a district-based model. Each operational health district (OD) covers 100,000 to 200,000 people and has health centres–each with a population of about 10,000 to 20,000 people–providing first level health care services and a referral hospital providing second (or third) level health care. The study was carried out between March and May 2007 in rural areas of three ODs, namely Mongkol Borei in Banteay Meanchey province, Sotnikum in Siem Reap province and Kirivong in Takeo province. The three ODs were chosen on the basis of the following criteria: availability of information on poverty prevalence, availability of public and private health care providers, and presence of a Health Equity Fund (HEF). The latter is a third party purchasing mechanism to identify the poor and pay user fees and other access-related costs on their behalf [16]–[18]. Table 1 summarises relevant characteristics of the study ODs.

**Table 1:** Relevant characteristics of the chosen ODs.

Characteristics	Mongkol Borei	Sotnikum	Kirivong
Total population	239,713	259,927	216,326
Population living below the poverty line*	38%	76%	35%
Number of health centres	19	21	20
Referral hospital	Provincial hospital with 195 beds, providing third level care	District hospital with 100 beds, providing second level care	District hospital with 80 beds, providing second level care
Outpatient care for chronic diseases at the referral hospital	Free HIV/AIDS-related care, supported by national programme	Free HIV/AIDS-related care and care for some chronic diseases, supported by national programme and NGO	Free HIV/AIDS-related care, supported by national programme
Public and NGO health facilities at the provincial town	No	Provincial hospital Two free NGO paediatric hospitals about 30 km away	Provincial hospital at 40 km away
Private health providers in the district	Many informal and formal practices Several high profile private clinics with operation theatre	Many low profile private practices Some higher profile clinics in Siem Reap town	Many low profile private practices (private practices by hospital personnel were restricted)
Health Equity Fund	At the referral hospital and few health centres All with pre-identification, and NGO operator	At the referral hospital and few health centres Approx. 1/3rd with pre-identification, and NGO operator	At the referral hospital and all health centres All with pre-identification, and pagoda-based operator
Households identified as eligible for Health Equity Fund**	30%	30%	21%

*Source: Estimation of Poverty Rate at Commune Level in Cambodia 2002, Ministry of Planning and WFP.

**Estimation based on results of recent identification of poor households conducted in these areas.

### Study design and data collection

We conducted a Rapid Household Survey (RHS), a cross-sectional household survey aimed at identifying households with a member suffering from a ‘major illness’ for further in-depth interviews. The sample size calculation was based on the expected incidence of major illness over one year (estimated at 5% of the households). To obtain 100 cases with a major illness, 2,000 households per OD were sampled, amounting to 6,000 households in total for the three ODs.

A sampling frame was constructed using the database of villages (2006–2007) from the National Institute of Statistics and the Ministry of Health's Health Coverage Plan [19]. A two-stage stratified cluster sampling design was adopted to randomly select the households. Each OD was sampled individually and a list of all villages with respective population figures was developed. When a village consisted of more than 250 households, it was divided into segments with at most 250 households per segment. From this list, 80 villages or clusters were randomly selected using simple random sampling without replacement. In each selected village all households were listed and 25 selected using linear systematic sampling. Weights (inversely proportional to the sampling probability) were calculated and integrated in the database. In Sotnikum villages were further stratified into those with and without pre-identification of poor households (i.e. a procedure through which a HEF card is issued to each eligible household).

The RHS questionnaire was developed in English (See file S1) and then translated into Khmer and tested in poor communities near the study sites. All research partners were involved in the questionnaire development to ensure consensus and to determine whether the questions reflected the reality in poor rural areas. Information was collected on household characteristics and their members, illness episodes affecting household members during the previous month, perceived serious illnesses (defined as those potentially impacting household livelihoods) during the year preceding the interview, related health seeking behaviour and health expenditures, and knowledge about and use of schemes intended to support those suffering from ill-health. Information on household assets commonly used for constructing socio-economic status indices [20] (housing condition, ownership of agricultural land, livestock, means of transport, and entertainment materials) was also collected to enable classification along socio-economic quintiles by use of Principal Component Analysis.

The survey took place over a period of two months. An ‘exploratory’ team went to the village one day prior to the survey to inform the village chief about the research, draw a village map and select the households. The map included the sampled households, the distance to the nearest district hospital and the presence of health centres and private health care providers. The exploratory team then met with the enumerators to hand over the map and discuss issues which needed to be addressed. Experienced enumerators with one week of training administered the questionnaire to the head of each selected household after a verbal consent was obtained. Often the spouse and other members were also present. Each interview lasted around 45 minutes.

To collect information on perceived serious illnesses, respondents of each household were asked to report all household members affected by illness or injury during the previous 12 months, where that illness had caused substantial concern in terms of individual's health or the potential financial consequences for the respective household. These include severe or life threatening conditions, chronic or recurrent conditions that resulted in some degree of disability or imposed care burdens on other household members, and conditions that required hospitalisation and/or spending a lot of money for treatment. For such conditions, the main symptoms and (eventual) diagnosis were recorded and a check was made as whether this diagnosis was made by a professional health provider. If a household member reportedly suffered from more than one serious illness, only the illness diagnosed by a professional health provider and with high treatment costs was considered.

### Data entry and analysis

Data were entered into a database format by trained persons and then cleaned and analysed by the authors with the help of a statistician, using SPSS 16.0 for windows. An asset-based principal component analysis was performed to construct household socio-economic status (SES) indices. Based on the household index score, household members were then categorised into quintile groups.

The reported diagnoses of perceived serious illness were checked, recoded and grouped by two medical doctors with public health experience. Some rather vague diagnoses were redefined based on reported symptoms and after consultation with the enumerators. In a next step, serious illnesses with clear diagnoses were then classified as ‘chronic lifelong conditions’ or ‘acute health problems’, while reported conditions for which the given information was insufficient (to be classified as chronic lifelong conditions or acute health problems) were labelled ‘non-specified conditions’.

Self-reported serious illnesses as percentage of survey households and household members were computed. Household members were stratified by groups of diagnoses, asset-ownership SES, gender and age, and proportions were compared using a Chi-square test. Significance was determined at the 5% level (p<0.05). Means of normally distributed data between two groups were compared, using the Independent-Samples t-test. For skewed data, a non-parametric test (Mann-Whitney) was applied.

### Ethical considerations

This study received ethical approval from the Cambodian National Ethics Committee on 14 February 2007, with reference number 002 NECHR. In rural Cambodia, the majority of people are illiterate. Written consent is not common practice and may send the wrong signal of weak or altered confidentiality. Therefore, a consent form to obtain verbal consent from respondents was proposed and approved by the Ethics Committee together with the study protocol. Prior to the interview, our enumerator read carefully the consent form. This consent form contains information on the objectives of the study, the selection process, risks, benefits and freedom of the participation, as well as information on confidentiality.

## Results

### Characteristics of survey households and household members

Table 2 presents key characteristics of survey households and respective members by OD. In total, 5,975 rural households comprising 33,161 members were visited. In Kirivong, 25 households in one selected remote village at the Vietnamese border could not be reached for interview, as they had temporarily migrated to Vietnam for work. Selecting another nearby village for replacement was not possible.

**Table 2:** Key characteristics of survey households and household members.

Variables	Mongkol Borei	Sotnikum	Kirivong	All districts
Total number of households	2,000	2,000	1,975	5,975
Total household members	11,495	10,950	10,716	33,161
% of households by size				
Households with 1-2 members	1.6	1.7	2.3	1.9
Households with 3-5 members	32.9	37.5	36.5	35.6
Households with 6-8 members	45.3	45.9	49.1	46.7
Households with ≥9 members	20.2	14.9	12.1	15.8
Average household size	5.7	5.5	5.4	5.5
Male-female sex ratio	0.99	0.93	0.95	0.96
% of household members by age group				
0-4 years	8.5	9.1	8.0	8.5
5-14 years	23.8	22.8	22.8	23.2
15-24 years	25.1	25.5	25.9	25.5
25-44 years	24.0	25.3	24.1	24.5
45-64 years	13.8	13.2	14.1	13.7
65- years	4.8	4.1	5.2	4.7
Mean age (year)				
Male	25.0	24.3	25.5	24.9
Female	27.3	26.8	27.7	27.2
% of household members aged >14 years by highest level of education				
None/primary incomplete	65.2	75.3	57.1	65.9
Primary	22.8	16.2	28.0	22.4
Secondary	7.2	4.5	10.6	7.4
High school	2.0	1.7	2.3	2.0
% of household members aged >14 years able to read a newspaper				
Yes	63.8	51.8	65.1	60.3
No	36.2	48.2	34.9	39.7
% of household members by employment status				
Employed	58.9	62.7	59.0	60.2
Student	26.7	24.6	29.7	27.0
Unemployed/stay home/household tasks	13.2	11.4	10.7	11.7
Retired/unable to work/monk	1.2	1.2	0.6	1.0
Poorest quintile (n = 6,540)	15.2	24.6	19.7	19.8
2 (n = 6,711)	16.8	21.7	22.6	20.2
3 (n = 6,608)	20.5	19.8	19.5	20.0
4 (n = 6,636)	22.1	18.8	19.0	20.0
Richest quintile (n = 6,615)	25.4	15.0	19.2	20.0

Most households (82%) had 3-8 members. In Mongkol Borei, there were significantly more households with 9 or more members (20%) than in Sotnikum and Kirivong (15% and 12% respectively). The average household size was similar in the three ODs with an overall average of 5.5 members, relatively bigger than the national average of 4.7 [8]. The age and sex structure of the survey population was similar to that of the Cambodian population [21].

The sample included slightly more women than men. The average male to female sex ratio was 0.96. The highest sex ratio was found in Mongkol Borei (0.99). The mean age for male and female was 25 and 27 years respectively, and was similar in the three ODs. The male and female mean age difference was statistically significant (Independent-Samples t-test; p<.001).

On average, 60% of the survey population aged 15 years or more were able to read a newspaper compared to national average of 78% [21] and 10% had a secondary or higher education. Comparison of the highest level of education and literacy rate among adults over 14 in the three ODs reveals that Sotnikum has the lowest education level and literacy rate in the sample. According to the distribution of household members by socio-economic quintile, Sotnikum was the poorest OD and Mongkol Borei was the richest one.

### Self-reported serious illnesses

Of the total of 5,975 visited households, 1,614 (27%) reported at least one member with a serious illness in the last twelve months. Table 3 provides the distribution of percentages of self-reported ‘serious illnesses’ by diagnosis. In total, 4,992 (15% of the total 33,161 household members) were reported to have a serious illness. Of those with a serious illness, 89% reported a diagnosis given by a professional health provider, while the remaining 11% received no diagnosis. Many diagnoses were not very specific, and were termed as a symptom or syndrome such as ‘abdominal pain’, ‘fatigue’, and ‘fever’. Chronic lifelong conditions and acute health problems accounted for 20% and 35% respectively of all the reported serious illnesses, while all non-specified conditions represented 33%.

**Table 3:** Frequency and percentages of self-reported serious illnesses by diagnosis.

Diagnosis	Frequency	% of all reported serious illnesses n = 4,992	% of all household members n = 33,161
Hypertension *	399	7.99	1.20
Typhoid fever †	357	7.15	1.08
Other lung and respiratory diseases	349	6.99	1.05
Physical injury †	299	5.99	0.90
Unknown abdominal pain	299	5.99	0.90
Tuberculosis †	254	5.09	0.77
Heart diseases *	253	5.07	0.76
Malaria †	223	4.47	0.67
Dengue †	181	3.63	0.55
Stomach ache	151	3.02	0.46
Urinary tract diseases	144	2.88	0.43
Chronic joint pain *	138	2.76	0.42
Acute respiratory infections †	122	2.44	0.37
Diarrhoea †	106	2.12	0.32
Gynaecological problems	102	2.04	0.31
Liver and bile diseases	97	1.94	0.29
Unknown fatigue	83	1.66	0.25
Pregnancy, delivery and complications †	70	1.40	0.21
Other intestinal disorders	67	1.34	0.20
Mental disorders *	62	1.24	0.19
Meningitis †	62	1.24	0.19
Skin diseases	57	1.14	0.17
Vitamin and other nutritional disorders	48	0.96	0.14
Haemorrhoids	48	0.96	0.14
Diabetes *	45	0.90	0.14
Unknown fever	42	0.84	0.13
Tumours and cancer *	41	0.82	0.12
HIV/AIDS *	36	0.72	0.11
Eye diseases	33	0.66	0.10
Ear – Nose – Throat (ENT)	33	0.66	0.10
Anaemia	33	0.66	0.10
Tetanus †	32	0.64	0.10
Food poisoning †	28	0.56	0.08
Hernia	25	0.50	0.08
Septicaemia †	16	0.32	0.05
Appendicitis †	15	0.30	0.05
Goitre *	14	0.28	0.04
Hemiplegia *	12	0.24	0.04
Osteoporosis *	4	0.08	0.01
Measles †	4	0.08	0.01
Epilepsy *	3	0.06	0.01
Others	53	1.06	0.16
No diagnosis	552	11.06	1.66
All diagnoses	4,992	100.00	15.05

‘*’*Chronic lifelong conditions;*

‘†’*Acute health problems*.

The ten most reported diagnoses, accounting for 55% of all reported serious illnesses, were hypertension, typhoid fever, other lung and respiratory diseases, physical injury, unknown abdominal pain, tuberculosis, heart diseases, malaria, dengue, and stomach ache. Hence, a large proportion of these top ten diagnoses were common tropical infectious diseases (typhoid fever, tuberculosis, malaria and dengue) and chronic lifelong conditions (hypertension and heart diseases). Physical injuries, which mainly resulted from road traffic accidents, were the fourth most common diagnosis. Stomach ache described by respondents as an unknown chronic pain in epigastria, featured also among the top ten diagnoses.

### Self-reported serious illnesses by socio-economic status and educational level

Table 4 provides an overview of the percent distribution of self-reported serious illnesses by group of diagnoses and SES. For all diagnostic groups, the proportion of household members reporting an illness declines from 17% in the poorest quintile to 13% in the richest quintile. This difference is statistically significant (p<.001). In general, there were significantly more poor household members reporting a serious illness (in comparison with rich household members). This was particularly the case for acute health problems, non-specified conditions and undiagnosed conditions. However, chronic lifelong conditions were more mentioned by the richest quintile than by the poorest one. The difference in percentage between the poorest and richest quintiles was statistically significant for all individual groups of diagnoses (p<.001).

**Table 4:** Percent distribution of self-reported serious illnesses by diagnostic and socio-economic groups.

Asset-based socio-economic quintiles	Chronic lifelong conditions	Acute health problems	Non-specified conditions	No diagnosis	All diagnostic groups
Poorest quintile (n = 6,540)	2.35	6.65	5.52	2.69	17.22
2 (n = 6,711)	2.98	6.20	5.10	2.21	16.48
3 (n = 6,608)	3.33	5.42	4.98	1.29	15.01
4 (n = 6,636)	3.04	4.61	4.88	1.24	13.77
Richest quintile (n = 6,615)	3.43	3.82	4.60	0.89	12.74
All the five quintiles (n = 33,110)*	3.03	5.34	5.01	1.66	15.04

*For 51 household members data were missing to calculate SES.

**Figure 1:**
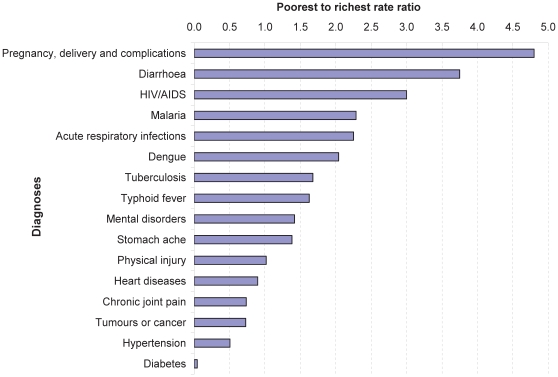
Rate ratio of self-reported serious illnesses in the poorest quintile to those in the richest quintile.

To assess the frequency of self-reported serious illnesses by SES, we calculated the rate ratio of some commonly reported conditions with specified diagnoses for the poorest quintile to the richest quintile (poorest-richest rate ratio; Figure 1). Certain illnesses, especially the common tropical infectious diseases, were considerably more frequent among the poor: ‘complications of pregnancy and delivery’ were five times more common in the poorest quintile than in the richest quintile. Diarrhoea and HIV/AIDS were respectively about four and three times more common, while malaria, acute respiratory infections and dengue were about twice more common in the poorest quintile compared to the richest quintile. Tuberculosis, typhoid fever, mental disorders, and stomach ache were about 50% more frequent in the poorest quintile. Conversely, a few illnesses (mainly chronic lifelong conditions such as heart diseases, chronic joint pain, tumours and cancer, hypertension and diabetes) were reported more frequently among household members in the richest quintile. Diabetes was reported far less by the poorest quintile. The frequency of physical injury was similar in the two quintiles. See file S2 for the frequency distribution of self-reported serious illnesses by diagnosis and socio-economic quintile.

Education can influence the lifestyle, health seeking behaviour and thus the health status of people. For example, the correlation between the mother's educational level and children's health has been well-established. To explore the correlation between educational level and self-reported illness in our sample, we compare the proportions of self-reported serious illnesses by diagnostic groups across educational levels of household heads, for all socio-economic groups and for the poorest and richest quintile only (Table 5). For all socio-economic groups together, there is no significantly different proportion of self-reported serious illnesses across educational levels of household heads. A similar pattern is also observed for the poorest and richest quintiles. However, the proportion of self-reported serious illnesses among household members in the poorest quintile is for all educational levels significantly higher than in the richest quintile. This reflects the correlation between the proportion of self-reported serious illnesses and SES presented in Table 4. But the correlation between SES and educational level of household heads is relatively weak (Pearson's r = .22, n = 5,714, p<.001).

**Table 5:** Percent distribution of self-reported serious illnesses by diagnostic group and educational level of household heads.

	Chronic lifelong conditions	Acute health problems	Non-specified conditions	No diagnosis	All diagnostic groups
All socio-economic groups					
None/primary incomplete (n = 24.151)	2.99	5.42	4.88	1.81	15.10
Primary (n = 5,002)	2.66	5.14	5.70	1.48	14.97
Secondary (n = 2,062)	3.64	5.58	4.85	1.02	15.08
High school (n = 407)	3.19	5.16	5.41	0.74	14.50
Primary and above (n = 7,471)	2.96	5.26	5.45	1.31	14.98
Poorest quintile					
None/primary incomplete (n = 5,455)	2.46	6.69	5.33	2.75	17.23
Primary (n = 737)	1.90	5.97	5.43	2.58	15.88
Secondary (n = 156)	1.28	9.62	7.05	2.56	20.51
High school (n = 26)	3.85	0.00	19.23	0.00	23.08
Primary and above (n = 919)	1.85	6.42	6.09	2.50	16.87
Richest quintile					
None/primary incomplete (n = 3,809)	3.49	3.44	4.67	1.05	12.65
Primary (n = 1,309)	2.37	4.89	3.97	0.84	12.07
Secondary (n = 871)	3.67	4.36	5.51	0.46	14.01
High school (n = 187)	3.74	4.28	5.35	0.53	13.90
Primary and above (n = 2,367)	2.96	4.65	4.65	0.68	12.93

### Self-reported serious illnesses by gender and age group

Table 6 shows the percent distribution of self-reported serious illnesses by diagnostic group, broken down by gender and age groups. Significantly more females than males reported a serious illness (16.7% vs. 13.4%; p<.001). Chronic lifelong conditions were twice as common among females (4.3%) than among males (1.8%) and the difference was observed in all age groups. There was a U-shape trend in the respective percentages of household members affected by a serious illness among different age groups. A relatively high percentage of the children under 5 suffer from a serious illness; then the percentage decreases up to the age group of 25-44 years; beyond this age group the percentage gradually increases again to reach the highest level among those aged 65 years or more (38% for males and 45% for females). We found a similar trend for acute health problems, non-specified conditions and undiagnosed conditions. Unsurprisingly, chronic lifelong conditions were more frequently reported with increasing age (by both gender groups) going from 0.1% for males and 0.2% for females under 5 to respectively 13% and 20% for males and females aged 65 years or above.

**Table 6:** Percent distribution of self-reported serious illnesses by group of diagnoses, by gender and age groups.

Gender and age group	Chronic lifelong conditions	Acute health problems	Non-specified conditions	No diagnosis	All groups
Females					
0–4 years (n = 1,421)	0.21	8.94	5.28	1.55	15.97
5–14 years (n = 3,755)	0.43	3.86	2.21	0.64	7.14
15–24 years (n = 4,124)	0.80	3.52	2.57	0.78	7.66
25–44 years (n = 4,135)	4.01	5.56	7.38	1.52	18.48
45–64 years (n = 2,587)	12.28	6.18	9.81	4.02	32.29
65- years (n = 920)	20.00	7.17	11.20	6.20	44.57
All females (n = 16,942)	4.25	5.15	5.47	1.78	16.65
Males					
0–4 years (n = 1,400)	0.14	10.79	5.93	1.79	18.64
5–14 years (n = 3,924)	0.33	4.59	1.81	0.76	7.49
15–24 years (n = 4,334)	0.55	3.83	1.98	0.81	7.18
25–44 years (n = 3,974)	1.79	5.69	5.84	1.46	14.77
45–64 years (n = 1,957)	4.91	6.75	9.51	3.38	24.55
65- years (n = 630)	12.86	6.51	12.70	5.71	37.78
All males (n = 16,219)	1.77	5.53	4.55	1.54	13.39

**Figure 2:**
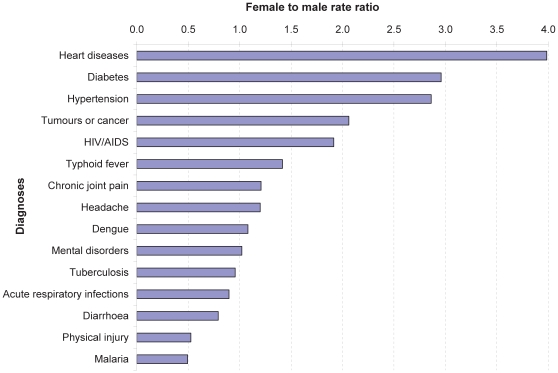
Female to male rate ratio of self-reported illnesses.

Figure 2 presents the female to male rate ratio of some commonly reported serious illnesses with specified diagnoses. Certain illnesses, mainly chronic lifelong conditions, were considerably more common among women than men. ‘Heart diseases’ were four times more frequently reported by women than men, followed by diabetes and hypertension (three times), while tumours or cancer and HIV/AIDS were twice as frequent. A few acute health problems, such as malaria, physical injury, and diarrhoea, were more frequently reported by men.

In the total survey sample, there were 2,821 children under 5 and 1,550 elderly aged 65 years or more. Table 7 shows that the ten most frequently reported diagnoses among young children were mainly common tropical infectious diseases such as acute respiratory infections, diarrhoea, dengue, typhoid fever, tuberculosis, malaria and meningitis, whereas for the elderly aged 65 years or more, chronic lifelong conditions were more frequent. However, acute health problems, such as tuberculosis and diarrhoea, featured also among the top ten diagnoses reported by the elderly.

**Table 7:** Top ten diagnoses among children under five and elderly aged 65 years or more.

No.	Top ten diagnoses among children <5 years	% of all household members; n = 2,821	Top ten diagnoses among elderly aged > = 65 years	% of all household members; n = 1,550
1	Other lung and respiratory diseases	3.93%	Hypertension	10.39%
2	Acute respiratory infections	2.09%	Tuberculosis	3.29%
3	Diarrhoea	2.06%	Heart diseases	2.58%
4	Dengue	1.84%	Other lung and respiratory diseases	2.39%
5	Typhoid fever	1.35%	Chronic joint pain	2.39%
6	Tuberculosis	1.03%	Physical injury	2.32%
7	Malaria	0.46%	Unknown abdominal pain	1.29%
8	Other intestinal disorders	0.46%	Unknown fatigue	1.16%
9	Meningitis	0.46%	Urinary tract diseases	1.03%
10	Unknown fever	0.39%	Diarrhoea	0.77%

**Figure 3:**
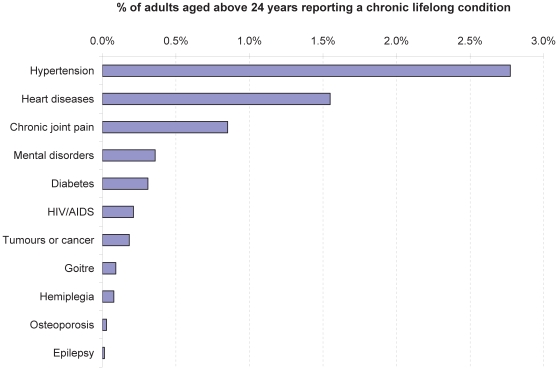
Chronic lifelong conditions reported as serious illness among adults aged above 24 years.

Figure 3 shows the percent distribution of chronic lifelong conditions reported as serious illness among adults aged above 24 years. Hypertension was the most frequently reported (by about 3% of the adults over 24), followed by heart diseases and chronic joint pain (2% and 1% respectively). Diabetes, AIDS and other chronic lifelong conditions were much less common (percentages of less than 0.5%).

### Self-reported serious illnesses and inpatient care

Of the 4,992 household members with a serious illness, only 1,482 (30%) said they had received inpatient care (defined as lying in a hospital bed for more than 24 hours). Acute health problems often led to inpatient treatment (40%), and were followed by non-specified conditions (29%) and chronic lifelong conditions (22%). The lowest percentage was found among household members with no diagnosis (12%). Socio-economic groups did not differ in terms of inpatient care. However, significantly more men than women received inpatient care (33% vs. 27%; p<.001).

## Discussion

This survey aimed at identifying households with a ‘major illness’ among the rural Cambodia's population for further in-depth investigation. We defined a major illness as a condition that potentially caused serious damage to the household livelihood strategies with increased risk of impoverishment. We identified these households by asking household heads (or spouses) to report all household members affected by a serious illness or injury that caused a major health or financial burden for their household, and this through probing for illnesses that were severe, life threatening, chronic, recurrent, or required hospitalisation and/or spending a lot of money for treatment. This indicates that the self-reported serious illnesses in this study include both clinically and economically defined serious illnesses, which may not necessarily be major illnesses. They are subject to a number of limitations, including recall and selection biases, commonly found in interview-based health surveys [22]–[25].

As for possible recall bias, the respondent may indeed not accurately remember the illness history of all household members. But this recall bias is likely minimal due to the fact that we focused on ‘serious illnesses’. The probability to remember such events is considerably higher than for mild diseases. Also, the data collection was carried out by a group of experienced and well-trained enumerators. Selection bias should also be limited given the random sampling procedure and the relatively big sample size. The sex and age structure of our survey population was similar to that of the Cambodian population.

The probability of reporting a perceived serious illness does not only depend on its incidence or prevalence in the survey population, but also on the respondent's awareness and perception of its seriousness. The seriousness of an illness can be differently assessed according to clinical and economical perspectives respectively. In general, poor people experience illness more often than the better-off and are more used to discomfort caused by illness than the latter. A recent study in Chad highlights that poor households often try to ignore health problems and absorb them into the experience of everyday life [26]. This suggests that from a clinical perspective many illnesses, especially chronic conditions, which are considered serious by the better-off, can be ignored or are considered minor by the poor. On the contrary, from an economical perspective, an illness that can be effectively treated, even at a relatively high cost (e.g. because it needs hospitalization), can be considered minor by the better-off, but may be perceived as serious by the poor, who are economically more vulnerable.

Many chronic diseases are often asymptomatic for many years; sufferers may not be aware of their condition. The fact that many survey household members with a serious illness–especially the poor–failed to get a diagnosis or got a non-specified diagnosis suggests their rather limited access to quality diagnosis, which may in turn result in restricted awareness of conditions or in wrong diagnoses. Pilsczeck observed that the quality of diagnostic procedures was limited even in a well-equipped and staffed hospital in Cambodia [27]. However, the somewhat limited accuracy of diagnosis does not jeopardize the aim of this study, as the health seeking behaviour of patients is usually driven by the way they perceive their disease, rather than by the label of the diagnosis. Several studies on economic consequences of illness were based on reported or perceived illness [3], [4]. This suggests that despite some limitations in terms of the method used, careful analysis and interpretation of the findings could still yield useful information for further in-depth study on socio-economic consequences of major illnesses on households.

Twenty seven percent of the survey households had at least one member with a serious illness in the year preceding the survey and 15% of the survey household members reported having at least one serious illness. This figure is much higher than our initial estimate of 5%, the hospitalisation rate in Cambodia. Yet, it is difficult to judge whether this figure is indeed high, as there is no reliable and comparable reference on the frequency of self-reported serious illnesses among the Cambodian population. A study in 2002 by Kenjiro in two rural villages in Cambodia [9] showed that about 80% of the approached households had suffered from serious illnesses or injuries in the 10 previous years. Many of these households lost their land due to the related costs for care. A multi-country analysis by Xu and colleagues [28] found that the highest incidence of catastrophic health expenditure was 10.5% (in Vietnam). Although these studies are not necessarily comparable to ours, they do provide an idea about the proportion of households potentially affected by economic consequences of major illnesses. Our findings suggest that a considerable proportion of Cambodian households are affected by serious illnesses that can threaten their livelihoods. In other words, this is a major public health and development concern that deserves policy attention.

Common tropical infectious diseases (typhoid fever, tuberculosis, malaria and dengue), chronic lifelong conditions (hypertension and heart diseases) and physical injury featured among the ten most frequently reported diagnoses. This picture is similar to the diagnoses reported in the Cambodian Health Management Information System [29] and reflects the double burden of communicable and non-communicable diseases in developing countries [30]. Perhaps surprisingly, ‘stomach ache’ described by respondents as an unknown chronic pain in the epigastria, was also one of the top ten diagnoses. Hypertension was the most frequently reported condition, but was still reported less than its estimated prevalence. The same holds for several other reported chronic lifelong conditions. Less than 3% of the surveyed adults aged over 24 reported suffering from hypertension and only 0.3% reported diabetes, whereas the prevalence of these conditions was estimated at 12% and 5% respectively in rural areas during two epidemiological surveys [31]. Also HIV/AIDS reporting is considerably lower (0.1%) than the estimated adult prevalence of 0.9% [32]. This indicates that a considerable number of people suffer a chronic lifelong condition without actually being aware of it. King and his colleagues [31] reported that two thirds of people with diabetes and more than half of those with hypertension in Cambodia were unaware of their condition. And even if they were aware of it, they might not perceive it as serious, as many chronic diseases are often not severe or life threatening until they lead to complications, which often happens at a later stage of the disease. The stigma attached to HIV/AIDS may lead to underreporting, as it is often a challenge to get people living with HIV/AIDS disclose their status [33], [34]. Unlike for chronic lifelong conditions, the reported frequency of acute health problems tended to be higher than the estimated incidence rate. For example, about 0.8% of the survey household members reported suffering tuberculosis within the previous year whereas the estimated incidence rate was 0.5% [35]. This could be due to the fact that many of the reported tuberculosis cases, including the high proportion of smear negative diagnosed tuberculosis by an NGO hospital among children in Sotnikum, were not real cases of tuberculosis.

The frequency of self-reported serious illnesses was strongly associated with household SES, gender and age; 17% of household members in the poorest quintile reported serious illness versus 13% in the richest quintile. This statically significant poor-rich difference could be due to the higher risk of illness and vulnerability to health shocks among the poor, as poor people often have worse health and suffer more often from severe health problems than the rich do [1]. According to the poorest to richest rate ratio for self-reported serious illnesses, there was a considerable difference among socio-economic quintiles for some acute health problems. ‘Pregnancy, delivery and complications’ were about five times more frequently reported by women in the poorest quintile than by those in the richest quintile. Common tropical infectious diseases, such as diarrhoea, acute respiratory infections, malaria, dengue, tuberculosis, HIV/AIDS, and typhoid fever, were about twice more common among the poorest than among the richest. In addition to the poverty-related vulnerability to illnesses and their consequences, the higher fertility rate and lower access to obstetric care among poor women in Cambodia [8], [36] could also account for the poor-rich difference in reported ‘pregnancy, delivery and complications’. Conversely, diabetes and hypertension were more frequently reported by the rich. This contradicts a statement by the World Health Organization that poor people are much more likely to develop chronic diseases than the wealthy [37]. However, in Cambodia diabetes and hypertension appear to be more prevalent among the rich than the poor who usually have traditional lifestyles, as shown by the epidemiological surveys that reveal a relatively higher prevalence of diabetes and hypertension (11% and 15%) in richer communities than in the poorer ones (5% and 10%). Diabetes and hypertension could also be more underreported by the poor (compared to the rich) due to a lack of access to reliable diagnostic facilities. Our results indicate that 11% of cases with a serious illness failed to get a proper diagnosis. Such failure to get diagnosed was three times more common in the poorest than in the richest quintiles. Another reason for the lower proportion of these self-reported chronic diseases among the poor could be the fact that they did not consider the diseases as serious enough from a clinical perspective, even if they were aware of these conditions. Although better educated respondents may have better knowledge about chronic diseases and are thus more likely to report these conditions, findings from our analysis of the proportion of household members reporting serious illnesses across educational levels of household heads do not support this.

The results indicate that self-reported serious illnesses tended to be more frequently reported by women (17%) than men (13%), in particular for chronic lifelong conditions: the female to male rate ratio was 3–4 times higher among women than men for heart diseases, diabetes, and hypertension. This seems to contradict results from other studies which showed no difference by gender for diabetes and hypertension [31] and heart diseases [38], while the analysis of Cambodia Demographic and Health Survey 2005 data by Rodgers pointed out that elderly women were more likely to be sick than men [39]. A possible explanation for this high frequency of reported chronic lifelong conditions among women could be that women in our sample were proportionally older than men while the prevalence of chronic diseases correlates with age. However, women of all age groups reported more chronic lifelong conditions than men. Possibly adult women come more in contact with health care providers and have thus an increased likelihood to be diagnosed. Such diagnosis can be made during antenatal care visits when blood pressure is measured, although diabetes and other chronic conditions require diagnostic means that are sometimes only available at hospital level. According to enumerators, some respondents (mainly women) reportedly termed palpitations or anxiety as heart diseases. Some studies on the economic burden of diseases showed that households using inpatient care tend to have experienced catastrophic health expenditures more often than those using outpatient care [40], [41]. In this study, less than one third of household members reporting a serious illness said they had received inpatient care. This suggests that major illnesses may not necessarily be the ones needing inpatient care, as shown by a study in Vietnam in which the impoverishing health care costs were not expenses associated with inpatient care, but rather non-hospital expenditures [42].

Despite some limitations in methods, this study provides new information on the frequency of self-reported serious illnesses among the rural population in Cambodia. It serves as a basis for further in-depth investigation on ‘major illnesses’ and their economic consequences on poor households, which in turn can help policy makers to formulate appropriate interventions to protect the poor from the financial burden associated with ill-health. Our findings suggest that every year a considerable proportion of households in rural Cambodia have members suffering from serious illnesses. Such conditions tend to be concentrated among the poor, children under five, women, and the elderly. The findings also reflect the double burden of communicable and non-communicable diseases in Cambodia. The most reported conditions include common tropical infectious diseases, chronic diseases (hypertension and heart diseases) and road traffic accidents. Poor women frequently reported complications related to pregnancy and delivery as serious illnesses.

## Supporting Information

File S1 Rapid household survey questionnaire.
(0.16 MB PDF)

File S2 Frequency of self-reported serious illnesses by diagnosis and socio-economic quintile.
(0.08 MB DOC)
