# Supplementary material for: Self-Reported Serious Illnesses in Rural Cambodia: A Cross-Sectional Survey
Source: PLoS One. 2010 Jun 3;5(6):e10930. doi: 10.1371/journal.pone.0010930 (PMC2880606; doi:10.1371/journal.pone.0010930)
Supplement: File S1 — Rapid household survey questionnaire. (0.16 MB PDF) [file pone.0010930.s001.pdf]

Protecting the rural poor against the economic consequences of major illness

Rapid Household Survey

QUESTIONNAIRE

Household identifier

Serial number:................................

Area level 1	Name of province	
Area level 2	Name of OD (Operational Health District)	
Area level 3	Name of commune	
Area level 4	Name of village	
Household number	Name of head of HH	

Enumerator					ID number:...			
Supervisor								
Date								

Permission to come back in the next three months

Section A: POVERTY ASSESSMENT

I. How would you describe your household's situation in term of the following?
("Sufficient" means whatever the respondent consider to be their minimum needs)

1.a	Food	1. Not sufficient	2. Just sufficient	3. More than sufficient
2.b	Clothing	1. Not sufficient	2. Just sufficient	3. More than sufficient
3.c	Accommodation	1. Not sufficient	2. Just sufficient	3. More than sufficient

2a	Are there times of the year when your household in short of food?	1. Yes<br>2. No ***(If No go to Q3)***
2b	**If yes**, which months?_____________________(record number of months)	Number of months..............................

3	Compared to other households in your community, would you describe the income of your household as:	1. Less than most others<br>2. Similar to others<br>3. More than most others

II. Can you tell me if there are any of the following in the household?

4.a	Culivate land area	Square area:..........................	Hectar:....................................

	Items	Number	Estimated Cost *(in Riel)*
4.b	TV sets		
4.c	Radio		
4.d	Stereo		
4.e	VCR		
4.f	Battery		
4.g	Phone		
4.h	Gas		
4.i	Stove		
4.j	Sewing machine		
4.k	Generator		
4.l	Water pump		
4.m	Ricemill		
4.n	Tractor		
4.o	Senitrator (Kor Yon)		
4.p	Plough		
4.q	Harrrow		
4.r	Oxcart		

III. Does your household own....?

	**Livestock**	**Number**	**Estimated Cost *(in Riel)***
5.a	Chicken/duck		
5.b	Pig		
5.c	Goat		
5.d	Cow		
5.e	Horse		
5.f	Buffalo		

IV. What are the main materials of the wall in your house? *(record observation)*

6.a	Plastic/tent	1
6.b	Thatch/palm/bamboo	2
6.c	Wood	3
6.d	Mud	4
6.e	Bricks	5
6.f	Corrugated iron	6
6.g	Other (specifies:..)	7

V. What are the main materials of the wall in your house? *(record observation)*

7.a	Plastic/tent	1
7.b	Thatch/palm/bamboo	2
7.c	Corrugated iron	3
7.d	Tiles/Cement/fibro cement	4
7.e	Other (specifies:..)	5

8. Is your household officially recognized as being poor?

1. Yes ..
2. No .. ***(If No go to Q9)***

If yes, by whom?____

Enumerator's assessment of poverty status of household compare to other?

1. Poor ..
2. Non-poor ..

Section B: DETAILS OF HOUSEHOLD MEMBERS

B1. Fof those who are living in this household at time of survey (prompt: sharing food):

No	B1	B2	B3	B4	B5	B6	B7
	Name<br>(Used only for identification during interview)	Age	Sex<br>1. Male<br>2. Female	Relation to household head<br>***(See code1)***	Main activity<br>***(See code 2)***	If age >14 Highest levcel of education Completed<br>1. None/primary incomplete<br>2. Primary<br>3. Secondary<br>4. High school<br>5. University<br>99. Don't know	If age >14 able to read a newspaper (in any language)?<br>1. Yes<br>2. No<br>99. Don't know
1							
2							
3							
4							
5							
6							
7							
8							

9							
10							
11							
12							
13							
14							
15							

For code 1 *(Relation to household head)*

1. Head 2. Spouse 3. Son/daughter 4. Son/daughter-in-law 5. Grandchild 6. Parent 7. Parent-in-law 8. Brother/sister 9.Niece/nephew by blood 10. niece/nephew by marriage 11. other relatives 12. Adopted/step child 13. Other (Specifies........................)

For code 2 *(type of household members' main activity)*

1. Family farm 2. Agricultural wage labor 3. Fisherman 4. Common natural property collector 5. Government employee 6. Skill worker employee
7. Unskilled worker employee 8. Household task 9. Unemployed 10. Student 11. Retired 12. Constructin worker 13. Motor Dup driver
14. Organization employee 15. Private company employee 16. Self business 17. Too old/small/stay home 18. Other (Specifies................................)
99. Don't know

B2. For husehold members living away from home at time survey:

No	Name (Used only for identification during interview)	Age	Sex<br>1. Male<br>2. Female	Relation to household head ***(See code1)***	Main activity ***(See code 2)***	If age >14 Highest levcel of education Completed<br>1. None/primary incomplete<br>2. Primary<br>3. Secondary<br>4. High school<br>5. University<br>99. Don't know<br>NA=Under 14 years old	If age >14 able to read a newspaper (in any language)?<br>1. Yes<br>2. No<br>99. Don't know	Reasons away from home<br>1. Internal migrant labor<br>2. Externl migrant labor<br>3. Education<br>4. Live with relatives<br>5.Other................
1								
2								
3								
4								

DEATH IN AN ACCIDENT IN THE PAST 12 MONTHS

1. Have any household members died during the last year? 1. Yes 2. No ***(If No go to section C)***

If yes: causes of death____

Section C: DETAILS OF HEALTH PROBLEMS

Have any household members (living here or elsewhere) been sick in the last month? Yes: .. Members:**……….**No: ..

(If no one has been sick go to sectin D)

**C1**	**C2**	**C3**	**C4**	**C5**	**C6**
Name<br>(Used only for identification during interview)	Use number table B1 or table B2	Was the problem?<br>1. light<br>2. Moderate<br>3. Severe<br>4. Chronic	If sick: Diagnosis(if available) or main symptom<br>***(See code 3)***	If sick: Where did they seek care/treatment?<br>***(See code 4)***	If sick: How much in total did they spend on treatment?<br><br>999. Don't know<br><br>***(In Riel)***

For code 3 *(Type of illness)*

1. Malaria, 2. TB, 3. Diarrhea, 4. Dengue, 5. HIV/AIDS, 6. Hypertension, 7. Diabetes, 8. Emergency obstetric, 9. Disease of urinary system, 10. Heart diseases, 11. Cough, 12. Common cold or acute respiratory infection, 13. Stomachache, 14. Fever,15. Typhoid, 16. Joint pain, 17. Fatigue, 18. Physical injury, 19. Pleurisy, 20. Pneumonia, 21. Liver abscess, 22. Hypoglycemia, 23. Asthma, 24. Calcium deficiency, 25. Heart failure, 26. Pharyngitis, 27. Abortion, 28. Glaucoma, 29. Gastric ulcer, 30. Appendicitis, 31. Stillbirth, 32. Hemorrhage after delivery, 33. Kidney stone, 34. Hepatitis, 35. Cataract, 36. Intestinal tumor, 37. Anemia, 38. Goiter, 39. Pterygium, 40. Meningitis, 41. Tumor in kidney, 42. Dysentery, 43. High blood pressure or hypertension, 44. Paraplegia, 45. Cholera, 46. Normal delivery, 47. Other (Please specify................................), 99. Don't know.

For code 3 *(Type of health care facility)*

1. National hospital in Phnom Penh 2. Provincial hospital 3. District hospital 4. Health center 5. Health post 6. Outreach 7. Private facility 8. Pharmacy 9. NGO/religious facility 10. TBA/VHW 11. Drugstore/shop 12. Kru Khmer 13. Monk/religious healer 14. Did not seek care 15. NGO clinic 16. Private practitioner 17. Other (specifies:.......................................) 99. Don't know

Section D: MEMBERS WITH SERIOUS HEALTH PROBLEMS

Have any household members (living here and elsewhere) suffered from a serious illness or disability over the last year? **Yes** .. Number......... **No** ..

**D1.** (Used only for identification during interview)	**D2.** Use number in table ***B1 or table B2***	**D3.** What were the main symptoms? ***(Multiple answer)*** (Enter diagnosis)	**D4.** Did a qualified doctor give them a diagnosis? (If so, ***See code 3***) 99. Don't know	**D5.** Did they obtain out patient care or buy drug to treat themselves? 1. Yes 2. No ***(If No go to D7***	**D6.** ***If yes,*** what was the total cost over the year? 0=None 999=Don't know	**D7.** Were they prescribed drugs by a qualified providers? 1. Yes 2. No ***(If No go to D10)***	**D8** ***If yes,*** did they purchase drugs? 1. Yes 2. No 99. DK ***(If Yes and DK go to D10)***	**D9.** ***If no,*** why didn't they purchase drugs? 1. Cost 2. Drug not available 3. Other 99. DK	**D10.** Did they receive any inpatient treatment? 1. Yes 2. No ***(If No go to D13)***	**D11.** ***If yes,*** how many days did they spend as an inpatient *(in total days over the year)*	**D12.** ***If yes,*** where did they receive inpatient treatment? ***(See code 4)***	**D13.** Were they told by a qualified provider that they needed inpatient care urgently? 1. Yes 2. No ***(If No go to section E)***	**D14.** ***If yes,*** did they follow this advice? 1. Yes 2. No 99. DK ***(If Yes and DK go to section E)***	**D15.** ***If no,*** why didn't they follow this advice? e. Cost 2. Long distance 3. Other............. 99. DK

Section E: SUPPORT FOR MEMBERS WITH SERIOUS HEALTH PROBLEMS

Ask for each member identified in section D2 as having a serious health problem

**E1**<br>Name	**E2**<br>Use number in table B1 or table B2	**E3**<br>Did they require substantial care for extended period by other household members?<br>0. No<br>(Other wise enter total days in months)	**E4**<br>For those of working age: Were they unable to work normally for extended periods?<br><br>0. No<br>999= children under 14<br>(Other wise enter total days in months)	**E5**<br>Did they receive financial help from any official source, either government or NGO?<br>0. N0<br>1. HEF<br>2. CBHI<br>3. Exemption for user fee<br>4. Other:.......<br>***(Multiple)***	**E6**<br>Did they receive financial help from any other source outside the household?<br>0. No ***(if No go to section F)***<br>1. Family<br>2. Friend<br>3. Other.......<br>99. DK<br><br>***(Multiple)***	**E7**<br>If yes, roughly how much money did they receive?<br><br>***(In Riel)***<br><br>999. Not know the number	**E8**<br>Beside in cash what help did they receive?<br>0. No<br>1. Treatment<br>2. Travel<br>3. Food<br>4. Livestock<br>5. Other:...............

Section F: GENERAL SITUATION

1. Were there time last year you had severe financial problem?
   1. Yes 2. No ***(If No go to 3)***

2. ***If yes,*** how did you manage?

2.a	Reduced savings	1
2.b	Reduced expenditure on food	2
2.c	Remove children from school	3
2.d	Sold stored food	4
2.e	Sold household goods	5
2.f	Sold production tools/cattle	6
2.g	Sold livestock	7
2.h	Sold productive land/residential land	8
2.i	Borrowed from relatives/friends/others	9
2.j	Borrowed with interest (specifies:...................................)	1. Money lender<br>2. Credit institute
2.k	Sought additional paid work	10
2.l	Other (Specifies:.......................................)	

3. With respect to serious health problems, were there time last year you had severe financial problem?
   1. Yes 2. No ***(If No go to 3)***

4. ***If yes,*** how did you manage?

4.a	Reduced savings	1
4.b	Reduced expenditure on food	2
4.c	Remove children from school	3
4.d	Sold stored food	4
4.e	Sold household goods	5
4.f	Sold production tools/cattle	6
4.g	Sold livestock	7
4.h	Sold productive land/residential land	8
4.i	Borrowed from relatives/friends/others	9
4.j	Borrowed with interest (specifies:...................................)	1. Money lender<br>2. Credit institute
4.k	Sought additional paid work	10
4.l	Other (Specifies:.......................................)	

**5**	How would you rank the following in term of your household expenditure last year?	1. Highest 4. Lowest ***(1 is the highest)***
	Food	
	Clothing	
	Education	
	Health care	

6. Were there any serious health problem that not treated because of the cost? 1. Yes 2. No

Were there any serious health problem related to health problems but not on cost? ***If yes,*** what problems?<br>..<br>..<br>..	1. Yes<br><br>2. No

Section G: KNOWLEDGE ABOUT AND USE OF SUPPORT SCHEMES

1. Do you know about the following? Or (Do you know of any X that helps the poor people to get medical care?

1.a	User Fee exemption	1. Yes	2. No
1.b	Community based health insurance	1. Yes	2. No
1.c	Health Equity Fund	1. Yes	2. No<br>***(If No go to section H)***

2. Do you know the purpose of HEF? *(do not read the answers)* *(Multiple)*

2.a	Help the poor people pay for medical care	1
2.b	Pay for medical cost at health facility X or Y	2
2.c	Pay for transportation to go to the hospital Y	3
2.d	Pay for food for patient at the hospital Y	4
2.e	Don't know	99

3. Do you have HEF book or card? 1. Yes (Still have) 2. No ***(If No go to section H)***
3. Never but taken away *(go to q4)*

4. Do you know how to use the HEF card when you go to the hospital or health center? 1. Yes 2. No

**5**	Since you have this card or book, do you feel....	**Code**
5.a	Much better protect against future disease	1
5.b	Better protect but I 'm still concerned	2
5.c	The card does not change anything	3

6. Have you ever received support from HEF for hospitalization last year? 1. Yes 2. No

If not,* why? *(Multiple)

1. Not eligible
2. No one was sick
3. Not sure if get supported
4. No need of hospitalization
5. Don't know where it is used
6. Went to other hospital outside catchments area
7. Other
99. Don't know

Section H: HEALTH SERVICE PROVIDERS

Is there any woman in the household currently pregnant?   1. Yes   2. No

If yes, how many months...?

Section I: POLICY ANALYSIS

**1**	Who do you think is providing assistance to help the poor in getting access to public health care when they are sick in your community? ***(Multiple answer)***	**Code**
1.a	NGO	1
1.b	The government	2
1.c	Local community	3
1.d	The local authority	4
1.e	No one	5
1.f	Don't know	99
1.g	Other..................................	

**2**	Who do you think should be responsible for helping the poor in getting access to public health care?	**Code**
2.a	The local authority	1
2.b	NGO	2
2.c	The government	3
2.d	The community	4
2.e	No one	៥
2.f	Don't know	99
2.g	Other	

**3**	What do you think of government effort to help poor people to get access to health care?	**Code**
3.a	Taking great effort	1
3.b	Taking better effort	2
3.c	Taking moderate effort	3
3.d	Taking little effort	4
3.e	Not doing anything	5
3.f	Don't know	99
