# Supplementary material for: Self-Reported Serious Illnesses in Rural Cambodia: A Cross-Sectional Survey
Source: PLoS One. 2010 Jun 3;5(6):e10930. doi: 10.1371/journal.pone.0010930 (PMC2880606; doi:10.1371/journal.pone.0010930)
Supplement: File S2 — Frequency of self-reported serious illnesses by diagnosis and socio-economic quintile. (0.08 MB DOC) [file pone.0010930.s002.doc]

**Annex 2: Frequency of self-reported serious illnesses by diagnosi**s and socio-economic quintile

| Diagnoses | Asset-based socio-economic quintiles | | | | |
| --- | --- | --- | --- | --- | --- |
|  | Poorest | 2 | 3 | 4 | Richest |
| Hypertension | 52 | 84 | 77 | 83 | 103 |
| Typhoid fever | 78 | 89 | 73 | 69 | 48 |
| Other lung and respiratory diseases | 97 | 92 | 61 | 49 | 50 |
| Physical injury | 62 | 47 | 65 | 64 | 61 |
| Unknown abdominal pain | 50 | 57 | 62 | 59 | 70 |
| Tuberculosis | 57 | 62 | 61 | 40 | 34 |
| Heart diseases | 35 | 49 | 74 | 54 | 39 |
| Malaria | 64 | 54 | 39 | 38 | 28 |
| Dengue | 51 | 42 | 35 | 28 | 25 |
| Stomach ache | 29 | 31 | 32 | 38 | 21 |
| Urinary tract diseases | 28 | 24 | 25 | 38 | 28 |
| Chronic joint pain | 25 | 31 | 21 | 27 | 34 |
| Acute respiratory infections | 36 | 32 | 19 | 18 | 16 |
| Diarrhoea | 30 | 30 | 20 | 18 | 8 |
| Gynaecological problems | 25 | 20 | 22 | 18 | 17 |
| Liver and bile diseases | 18 | 22 | 24 | 17 | 16 |
| Unknown fatigue | 19 | 20 | 20 | 10 | 14 |
| Pregnancy, delivery and complications | 24 | 19 | 18 | 4 | 5 |
| Other intestinal disorders | 15 | 13 | 11 | 18 | 10 |
| Mental disorders | 17 | 11 | 12 | 10 | 12 |
| Meningitis | 10 | 13 | 14 | 12 | 13 |
| Skin diseases | 14 | 11 | 10 | 10 | 12 |
| Vitamin and other nutritional disorders | 9 | 11 | 4 | 11 | 13 |
| Haemorrhoids | 11 | 9 | 7 | 9 | 12 |
| Diabetes | 1 | 4 | 7 | 10 | 23 |
| Unknown fever | 12 | 4 | 11 | 8 | 6 |
| Tumours or cancer | 8 | 6 | 13 | 3 | 11 |
| HIV/AIDS | 9 | 4 | 11 | 7 | 3 |
| Eye diseases | 4 | 5 | 9 | 5 | 10 |
| Ear-Nose-Throat (ENT) | 3 | 2 | 11 | 7 | 10 |
| Anaemia | 7 | 7 | 7 | 10 | 2 |
| Tetanus | 8 | 10 | 6 | 3 | 5 |
| Food poisoning | 10 | 6 | 2 | 5 | 5 |
| Hernia | 7 | 4 | 6 | 6 | 2 |
| Septicaemia | 1 | 7 | 5 | 3 | 0 |
| Appendicitis | 1 | 5 | 1 | 3 | 5 |
| Goitre | 3 | 5 | 1 | 4 | 1 |
| Hemiplegia | 2 | 3 | 3 | 4 | 0 |
| Osteoporosis | 0 | 2 | 1 | 0 | 1 |
| Measles | 3 | 0 | 0 | 1 | 0 |
| Epilepsy | 2 | 1 | 0 | 0 | 0 |
| Others | 13 | 10 | 7 | 11 | 11 |
| No diagnosis | 176 | 148 | 85 | 82 | 59 |
| All diagnoses | 1,126 | 1,106 | 992 | 914 | 843 |
